# Algebraic Connectivity Control in Distributed Networks by Using Multiple Communication Channels

**DOI:** 10.3390/s21155014

**Published:** 2021-07-23

**Authors:** Karlo Griparić

**Affiliations:** Department of Engineering, University of Pula, Zagrebacka 30, 52100 Pula, Croatia; kgriparic@unipu.hr; Tel.: +385-52-377-084

**Keywords:** algebraic connectivity, distributed network, multiple communication channels, multi-agent systems, ad hoc communication

## Abstract

The effectiveness of collaboration in distributed networks, such as sensor networks and multi-agent systems, relies on nodes’ ability to exchange information. The availability of various communication protocols with different technical properties opens the possibility to guarantee connectivity during a system’s operation in any condition. A communication network can be represented by a graph on which connectivity can be expressed by a well-known algebraic connectivity value or Fiedler value. It is one of the most important tools used in many applications where connectivity preservation is required. In this paper, a trust-based consensus algorithm for algebraic connectivity estimation has been implemented. To guarantee the accomplishment of the global objective and the system’s performance, our contributions include: (i) a novel decentralized framework for combining multiple communication channels in a resulting channel and (ii) a decentralized algebraic connectivity control law that dynamically changes the number of agents in the system during operation. The proposed algebraic connectivity control strategy has been evaluated in simulations and in a real multi-robot system using two channels with different properties and initial topologies.

## 1. Introduction

Distributed networks have found huge potential in many applications, from sensor networks to multi-agent systems, where a group of simple and inexpensive nodes or agents should achieve a common goal in a distributed and parallel way. The advantages of such systems include scalability, robustness, and resistance to failures. Collaboration between agents is based on distributed algorithms, which define the procedure for information exchange in such systems, where their use is often based on the assumption of communication availability during the operation. However, in many applications, collaborating agents should have the ability to operate without external communication infrastructure, i.e., by using an ad hoc communication protocol, each agent is able to exchange information only with agents that are in its vicinity. On the other hand, information exchange between agents has a direct influence on the system’s performance including convergence speed, effectiveness of cooperation, and accuracy of achieving a global goal [1]. The first goal of our study was how to incorporate information about network availability into an agent’s local control law.

Wireless communication, implemented in various protocols, is the most widespread communication channel used in distributed networks, especially in multi-agent systems, in which agents change their positions over time. Variations in communication performances, caused by the dynamically changing topology and environment where the distributed network is operating, are difficult to predict, therefore making it difficult to guarantee the quality of communication. In the field of distributed multi-agent systems, substantial contributions have been made to increase network reliability. Early approaches that introduced an agent’s communication performance into its motion controller relied on the distance between nodes in the network. In some cases, this disc-based model is insufficient for preserving network connectivity during system operation. Aside from the mentioned distance-related signal quality relation, signal distortion is a common issue in wireless networks and includes phenomena such as small-scale and large-scale fading [2] and path loss [3]. The usage of distributed collaboration algorithms in networked systems with unstable communication is limited in applications where loss of connectivity does not have an influence on the system’s performance and goal achievement. However, when a distributed algorithm for collaboration between agents is applied, such as a consensus algorithm, the connectivity of the underlying communication network has a direct influence on the speed of convergence to a global goal [4,5]. In order to overcome the drawbacks of distributed wireless networks, the second goal introduced in this work was an algorithm that combines several communication channels into a single communication graph. Using multiple communication channels in order to improve the network performance of a multi-agent system has been motivated by the availability of many different communication protocols and their different properties. By combining the best available performance by means of speed, bandwidth, and energy consumption of a certain communication protocol, we tested the hypothesis that multiple communication channels contribute to the robustness of the communication network.

An additional motivation for combining multiple communication channels was found in the developed robotic system introduced in [6], where the authors, in the developed network of static robots for interaction with honeybees, integrated two communication channels, i.e., Ethernet local area network and infrared module, for communication with the nearest neighbours. The described multi-robot system uses a decentralized network control algorithm for coordinating each robot’s actions. The dynamical topology is not just a result of a variable number of links but also depends on the number of agents operating in the system. If an agent estimates the global algebraic connectivity with a fixed number of agents, it becomes obvious that the algebraic connectivity is equal to zero if one or more nodes fail. To cope with this graph’s property, the third goal of this paper was to provide an algorithm that dynamically changes the number of collaborating agents involved in the estimation of the algebraic connectivity. The trust-based consensus algorithm for the estimation of the topology of a communication graph has been used. According to a given criteria, the method calculates the resulting adjacency matrix based on two estimated adjacency matrices and consequently provides a graph with a larger spectrum of possible topology combinations that lead to an increased maximal value of algebraic connectivity.

To summarize, the problems investigated in this paper are related to:

- The incorporation of network availability information (Fiedler value) into the decision-making process of each agent in a decentralized manner.
- Signal disturbances in wireless communication protocols that limit the use of distributed algorithms in networked systems.
- A multi-agent system that is able to dynamically change the number of collaboration agents in a decentralized manner.

In the remainder of this paper, we first provide a literature overview in Section 2. In Section 3, we give basic notations on graph theory and preliminaries on the decentralized trust-based algorithm for algebraic connectivity estimation in multi-agent systems. Section 4.1 introduces a method for combining multiple communication channels. In Section 4.2, we propose algorithms for the connectivity maintenance of a communication network during operation in a dynamic environment. Section 5 presents simulation and experimental results that demonstrate the increased robustness and efficiency of the multi-agent system when a communication network with multiple channels is used. Finally, a discussion and conclusive remarks are given in Section 6.

## 2. Literature Overview

Increasing the performance of a decentralized multi-agent system has been intensively studied in the last decade, where significant progress has been made in the field of distributed control algorithms including algorithms for swarming [7,8,9], consensus [10,11,12], and rendezvous [13]. The ability of multi-agent systems to execute a certain task in parallel has been widely exploited in many applications, including logistics in autonomous warehouses [14], the exploration of unknown environments [15], manipulation [16,17], surveillance [18,19], search-and-rescue [20], interaction with animal species [21,22], and sensor networks [23,24,25]. The mentioned coordination algorithms have been applied in many different applications where different types of robots have been used, including unmanned ground, aerial, and underwater vehicles [26,27,28,29]. Additionally, similar communication and collaboration principles can be found in sensor networks used in various fields such as energy management in buildings [30], monitoring of large areas [31], industrial automation [32], and biomedical observation [33]. Collaborative wireless networks share many similarities with multi-agent systems, including limited processing capabilities, communication range, and modest energy storage of spatially distributed autonomous devices. Many scientifically interesting topics in the field of collaborative networks have been published in recent years, such as beamforming collaboration [34], the impacts of nodes’ special distribution to the effectiveness of collaboration [35,36], and synchronization protocols [37,38].

Preserving connectivity during the operation of multi-agent systems can be implemented using two approaches: an approach to maintain local connectivity and an approach to maintain global connectivity [39,40,41]. The local connectivity approach consists of maintaining communication links with agents that are available from the beginning of a system’s operation while the agent’s actions are performed to preserve initial links. The global approach is based on mathematical formalism where the communication network of a multi-agent system is described using graph theory. If a multi-agent system can be described with an undirected graph, then the second smallest eigenvalue of the corresponding Laplacian matrix, also called the algebraic connectivity or Fiedler value, represents the measure of communication network connectivity. Maximizing the algebraic connectivity in weighted undirected graphs represents an optimization problem. On the other hand, maximizing the value of the algebraic connectivity increases the computational complexity of the control algorithm and gives rise to information flooding in the information exchange process. Furthermore, a network topology dynamically changes due to the varying number of operating agents, errors, and failures in communication, as well as the changing distance between agents in the case of mobile agents.

The effects of communication channels fading in wireless networks on reaching consensus are presented in [42], while the sufficient conditions for channels’ performances that guarantee the convergence of the consensus algorithm are given in [43]. The authors in [44] provide a comprehensive overview of the multi-scale dynamics of wireless links for networked robotic systems. A more accurate probabilistic model that predicts signal quality during agent’s motion is provided in [45]. An intermittent connectivity framework for a multi-robot system is proposed in [46]. The authors consider a team of networked robots with limited communication capabilities, where connectivity between robots can be lost under some strict conditions. The presented methods to increase the stability of communication in ad hoc networks are computationally too complex for the single agents often used in multi-agent systems or wireless sensors networks.

## 3. Basic Notations

### 3.1. Graph Theory Notations

At the beginning of this section, we introduce the graph theory notations and basic concepts used in this paper. A graph G=(V,E) consists of a set of vertices V={1,2,…,n}, where *n* is the total number of vertices in the graph, and a set of edges E⊂V×V representing connections between vertices. The neighbours that are adjacent to vertex *i* are called the neighbours of *i*, and this set is denoted as $N_{i}=\left\{v_{j}\in V:e_{ij}\in E\right\}$.

There are various matrices that can be associated with a graph and reflect its properties. The adjacency matrix $A\in R^{n\times n}$ defines the connection of the vertices in the graph. The degree matrix $D=\mathrm{diag}(d_{1},\ldots ,d_{n})$ is a diagonal matrix where each element $d_{i}$ denotes a vertex *i* degree and is calculated as $\;d_{i}=\sum_{j=1}^{n}a_{ij}$, where non-diagonal elements are equal to zero. The Laplacian matrix is defined as L=D−A.

For an undirected graph, L is a symmetric matrix with real eigenvalues that can be ordered in a non-decreasing order:

$$\lambda_{1}\leq \lambda_{2}\leq \ldots \leq \lambda_{n}.$$

The second smallest eigenvalue of the Laplacian matrix, denoted by $\lambda_{2}$, is called the Fiedler value or algebraic connectivity and is a measure of connectivity of the associated graph. The graph is connected if and only if $\lambda_{2}>0$. The value of the algebraic connectivity is defined by the topology of a graph and the graph’s parameters, such as the number of vertices *n*, number of edges *m*, minimal degree $d_{min}$, etc. It is worth pointing out that for several popular graph topologies, such as complete, cycle, or cube graphs, the algebraic connectivity is known and can be determined by the number of vertices *n* in the graph. For instance, the algebraic connectivity of a complete graph is equal to $\lambda_{2}=n$, while for a cycle graph, it is equal to $\lambda_{2}=2\left(1-cos\frac{2\pi }{n}\right)$. However, the topology of a graph can be changed by allowing operations over the graph that may include the removal or addition of edges and vertices. Therefore, for an incomplete graph, the maximal value of the algebraic connectivity is upper bounded by the parameters of a graph. For example, the upper-bound of algebraic connectivity for an incomplete graph with *n* vertices is defined by

$$\lambda_{2}\leq n-2,$$

while the bound related to the minimal degree is given by the following inequality:

$$2d_{min}-n+2\leq \lambda_{2}\leq \frac{n}{n-1}d_{min}.$$

A comprehensive overview of the graph’s spectral properties can be found in [47].

### 3.2. Decentralized Algorithm for Algebraic Connectivity Estimation

Consider a multi-agent system with *n* agents in which the network topology can be described with the time-varying weighted undirected graph *G*, where the weight of the edges determines the quality of the communication link between two agents. Assume that each agent can exchange information only with agents that belong to the agent’s set of neighbours. In order to estimate an adjacency matrix in a distributed way, each agent *l* should compute its local estimate of the graph’s adjacency matrix $A^{l}$. We used a trust-based consensus algorithm, introduced in [48], given with the following discrete update law for the adjacency matrix element $a_{ij}^{l}$ of the agent’s *l*:

(1) $$a_{ij}^{l}\left(k+1\right)=a_{ij}^{l}\left(k\right)+\epsilon \times \Delta a_{ij}^{l}\left(k\right)$$

where $a_{ij}^{l}\in \left[0,1\right]$ is the agent’s *l* adjacency matrix element which defines the weight of the link that connects the agent *i* and *j*, k∈{0,1,2,...} is a discrete time step, and ϵ>0 is a period of discretization. The change of the state $\Delta a_{ij}^{l}\left(k\right)$ at each time step *k* is defined by Equation (2a,b).

Δaijl(k)={(2a)∑p∈Nlaipl(k)(aijp(k)−aijl(k))+(τijl(k)−aijl(k)),j∈Nl,(2b)∑p∈Nlaipl(k)(aijp(k)−aijl(k)),j∉Nl

where $N_{l}$ is a set of neighbours of robot *l*, $a_{ip}^{l}\left(k\right)$ is the (i,p) element of the agent’s *l* adjacency matrix, $a_{ij}^{p}\left(k\right)$ is the (i,j) element of the agent’s *p* adjacency matrix, and $\tau_{ij}^{l}\left(k\right)\in \left[0,1\right]$ is an observation function representing the ability of a communication channel between nodes *i* and *j*. The conditions under which the presented algorithm converges are given in the theorem presented in [49].

Assume that the performance of a communication link is defined by the number of exchanged packages between two nodes in a certain time period as defined by the following exponential observation function:

(3) $$\tau_{ij}^{l}\left(k\right)=e^{-K_{e}*\delta_{ij}^{2}\left(k\right)},$$

where $K_{e}$ is a constant that defines the rate of change of the exponential function, and $\delta_{ij}\left(k\right)$ refers to the ability of a communication link between agents *i* and *j* in a certain discrete time step *k*.

## 4. Theoretical Formulation

### 4.1. Multiple Communication Channels

The aim of any connectivity control law is to maintain a certain value of the algebraic connectivity during operation of a multi-agent system. Since the communication network in observed systems is usually based on the agents’ local capabilities, we proposed a method that combines multiple physical communication channels with different properties in order to improve the connectivity of the networked system.

**Figure 1:**
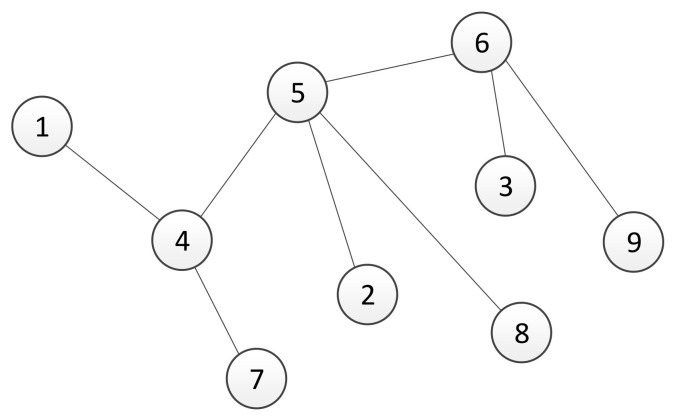
Graph topology of channel *A* of the system with 9 agents, where numbers represent identification numbers of agents.

**Figure 2:**
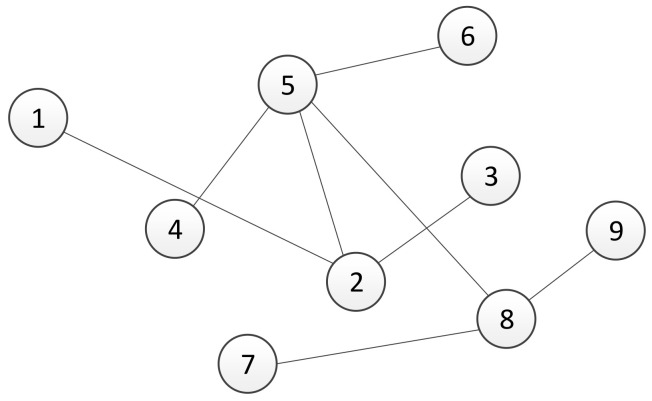
Graph topology of channel *B* of the system with 9 agents.

Assume that in the considered multi-agent system, there exists two different types of communication interfaces. Let *A* and *B* be adjacency matrices of the graphs for each communication channel, where the matrices’ elements are estimated using a trust-based consensus algorithm described in Section 3. In the rest of this section, we propose a method for combining different types of communication channels. For the sake of clarity, we have introduced our method using a system of nine agents with two physical channels, but it is not limited to that number of agents. Assume that communication protocols, denoted as *A* and *B*, are represented with a graph’s topologies shown in Figure 1 and Figure 2, respectively. Since each communication channel has a different topology, the question is how to construct a communication network by combining links from different channels. The resulting network, whose adjacency matrix is denoted by *T*, should include links with better quality among the available channels.

The weight of the adjacency matrix element estimated using a trust-based consensus algorithm represents the ability of a communication link between two nodes in the network. In addition, there exist many other physical parameters and phenomena that can influence the performance of information flow between connected agents. Furthermore, real multi-agent systems are unimaginable without ubiquitous wireless networks available in multiple communication protocols. Implemented on different physical layers in accordance with the OSI representation, the properties of the selected protocol may differ in bandwidth, distance between nodes, package delays, and power consumption. Additionally, in wireless networks, many phenomena of signal distortion may appear, including small-scale and large-scale fading and path loss, which may cause variability in the performance of the communication link. Therefore, we introduced a quality coefficient that represents the ability of each communication protocol, defined with the following equation:

(4) $$\delta_{ij}^{\left(A,B\right)}\left(k\right)=\frac{1}{K_{c}^{\left(A,B\right)}}\delta_{ij}\left(k\right)$$

where $\delta_{ij}\left(k\right)$ is a difference between transmitted and received packages between agents *i* and *j* at a discrete time step *k*, and $K_{c}^{\left(A,B\right)}$ is a quality constant of the communication channel *A* or *B*.

Each agent *l* computes a resulting local adjacency matrix $T^{l}$ by combining links with better quality from both available channels. Therefore, each element in the resulting matrix is equal to the adjacency matrix element with a higher value and can be written as

(5) $$t_{ij}^{l}=max\left(a_{ij}^{l},b_{ij}^{l}\right).$$

### 4.2. Dynamic Network

**Figure 3:**
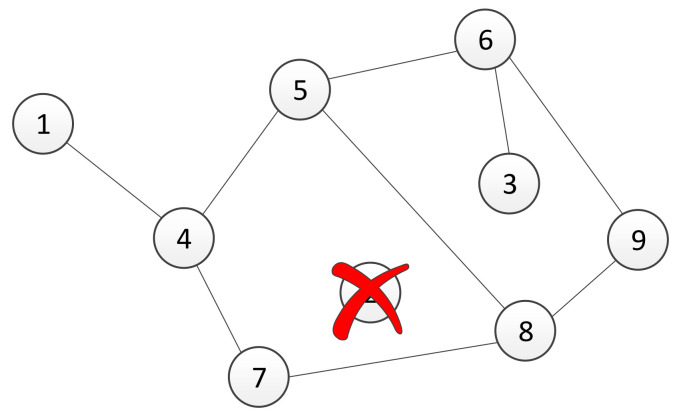
A graph of networked multi-agent system after failure of agent 2.

**Figure 4:**
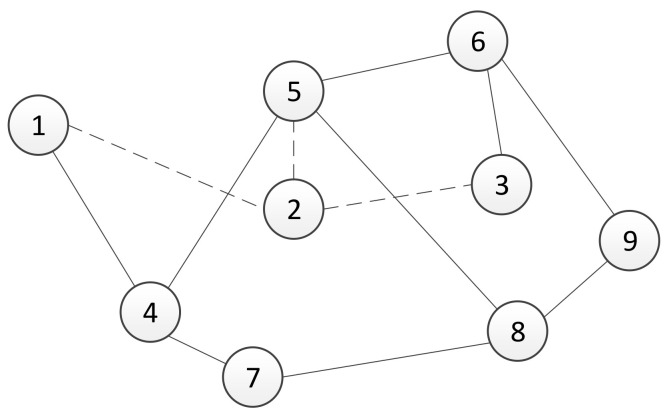
Network topology of the multi-agent system when a new agent is added. Solid lines represent a graph topology of 8 agents, while dashed lines represent established links of a new agent whose identification address is 2.

In this section, we will study the robustness of the used algebraic connectivity estimation algorithm under perturbations in the number of agents in the system. In a real multi-agent system, a loss of communication with one or more agents is typically caused due to the failure of an agent. When a decentralized trust-based consensus algorithm for the estimation of the algebraic connectivity of the communication network, described in Section 3, has been used, a failure or connectivity loss with a single agent causes an inability to control the connectivity of the remaining network. If the agents that are still operating calculate the algebraic connectivity using a fixed number of agents in the network, usually determined with an initial condition, then they are unable to increase its value. Specifically, in the case of agent failure, the graph’s adjacency matrix elements that represent its links are equal to zero. Thus, the corresponding Laplacian matrix has a zero row, and its second smallest eigenvalue is equal to zero as well. In order to overcome this problem, we present an algorithm that dynamically resizes a group of collaboration agents and ensures that the connectivity of agents with acceptable performance remains at a certain level. Basically, we have two possible cases here. The first case is when the performance of agents becomes too poor (Figure 3). In this case, the agent is affecting the system’s performance. Therefore, the algorithm should detect the critical agent and remove it from the group. The second case includes the moment when a new agent is added to the group (Figure 4). Without the loss of generality, the presented scenarios for removing and adding an agent are described using a system with nine agents. The size of the system is chosen because the effects of variation in group size can be easily formally written. Furthermore, the algorithms are written in a general form that allow for their implementation on any agent in the group of *n* agents.

Since we are dealing with decentralized agents, the proposed approach of dynamically changing the group size of collaborating agents cannot be directly implemented because of the decentralized properties of the algebraic connectivity estimation algorithm. Specifically, in the adjacency matrix, elements represent a weight of the link between two agents in the network. Therefore, removing an agent from the system means deleting a row and column from each local matrix at the same time step. Since this can not be guaranteed in decentralized systems, we proposed an algorithm that runs on each agent and detects the collaboration inability of an agent and removes it from future calculation. Additionally, we have proposed certain criteria for adding an agent in the existing multi-agent system.

In the algebraic connectivity estimation algorithm used in this paper, the adjacency matrix row and column number represents an identification number of a specific agent. The algorithm exchanges the elements of the algebraic matrices with neighbouring agents. Therefore, the algorithm expects that each element corresponds to the same link in the network. When a manipulation in the number of nodes in the graph is allowed, the adjacency matrix elements of neighbouring agents with some indexes can represent different links. To ensure that the adjacency matrix’s elements of two agents represent the same link, we introduced a mapping strategy by using an address vector on each agent. The vector M∈R, the size of which is equal to the number *n* of collaboration agents in the system, is defined as

$$M^{l}=\begin{array}{c} 1\\ \vdots \\ n \end{array}\left[\begin{array}{c} ID_{1}\\ \vdots \\ ID_{n} \end{array}\right]$$

where the *i*th row in the agent’s *l* adjacency matrix represents the identification number of the corresponding agent.

First, let us consider a case when an agent should be removed from a collective decision. Suppose that at a certain time step, a communication with an agent *m* fails. Because of the failure, on the remaining agents, the algebraic connectivity value will approach zero. The proposed strategy should detect an agent with low performance and remove its measurements from the estimation algorithm. To do this, each remaining agent *l* should check if its local estimate of the algebraic connectivity $\lambda_{2}^{l}\left(k\right)$ and its node degree $d^{l}\left(k\right)$ are within given thresholds defined with the following inequalities:

1. $\lambda_{2}^{l}\left(k\right)\leq \lambda_{2c}$
2. $d^{l}\left(k\right)\leq d_{c}$.

where $\lambda_{2c}>0$ is a constant that defines a critical value of the algebraic connectivity, $d_{c}>0$ is a constant that defines a threshold for minimal node degree, and *k* is a discrete time step. If the first condition is satisfied, then the agent *l* should check the second condition.

In order to demonstrate the effectiveness of the proposed algorithm, consider a network of n=9 agents where the agent’s *l* adjacency matrix $T^{l}$ with the corresponding agents’ identification numbers at the initial moment is equal to

$$\begin{array}{cc} \; & \;\;\;\;\;\;1\;2\;\;3\;\;\;\;4\;\;\;\;5\;\;6\;\;7\;\;8\;\;9\;\\ \; & T^{l}=\begin{array}{c} 1\\ 2\\ 3\\ 4\\ 5\\ 6\\ 7\\ 8\\ 9 \end{array}\left[\begin{array}{ccccccccc} 0 & 1 & 0 & 1 & 0 & 0 & 0 & 0 & 0\\ 1 & 0 & 1 & 0 & 1 & 0 & 0 & 0 & 0\\ 0 & 1 & 0 & 0 & 0 & 1 & 0 & 0 & 0\\ 1 & 0 & 0 & 0 & 1 & 0 & 1 & 0 & 0\\ 0 & 1 & 0 & 1 & 0 & 1 & 0 & 1 & 0\\ 0 & 0 & 1 & 0 & 1 & 0 & 0 & 0 & 1\\ 0 & 0 & 0 & 1 & 0 & 0 & 0 & 0 & 0\\ 0 & 0 & 0 & 0 & 1 & 0 & 0 & 0 & 0\\ 0 & 0 & 0 & 0 & 0 & 1 & 0 & 0 & 0 \end{array}\right] \end{array}$$

Now, suppose that at some time step, the agent with identification number 2 fails. A resulting communication graph is presented in Figure 3, where links with agent 2 no longer exist.

Therefore, after the period of transient response, the elements of all local adjacency matrices regarding agent 2 converge to zero, and the value of the agent’s *l* matrix is equal to

$$\begin{array}{cc} \; & \;\;\;\;\;\;1\;2\;\;3\;\;\;\;4\;\;\;\;5\;\;6\;\;7\;\;8\;\;9\;\\ \; & T^{l}=\begin{array}{c} 1\\ 2\\ 3\\ 4\\ 5\\ 6\\ 7\\ 8\\ 9 \end{array}\left[\begin{array}{ccccccccc} 0 & 0 & 0 & 1 & 0 & 0 & 0 & 0 & 0\\ 0 & 0 & 0 & 0 & 0 & 0 & 0 & 0 & 0\\ 0 & 0 & 0 & 0 & 0 & 1 & 0 & 0 & 0\\ 1 & 0 & 0 & 0 & 1 & 0 & 1 & 0 & 0\\ 0 & 0 & 0 & 1 & 0 & 1 & 0 & 1 & 0\\ 0 & 0 & 1 & 0 & 1 & 0 & 0 & 0 & 1\\ 0 & 0 & 0 & 1 & 0 & 0 & 0 & 0 & 0\\ 0 & 0 & 0 & 0 & 1 & 0 & 0 & 0 & 0\\ 0 & 0 & 0 & 0 & 0 & 1 & 0 & 0 & 0 \end{array}\right] \end{array}$$

The local value of the algebraic connectivity is calculated from its corresponding adjacency matrix and is equal to zero, i.e., $\lambda_{2}^{l}=0$. Note that all elements regarding agent 2 are equal to zero, and its node degree is also equal to zero $d_{2}=0.$ Accordingly, both conditions about the critical agent are satisfied. Thus, each agent *l* should construct a new adjacency matrix by removing the row and column that represent the critical agent. In the given example, agent 2 is the critical agent, and a new matrix should not contain a row and column that represent that agent. Illustratively, the row and column of the adjacency matrix that should be removed are struckthrough as presented in the following matrix:


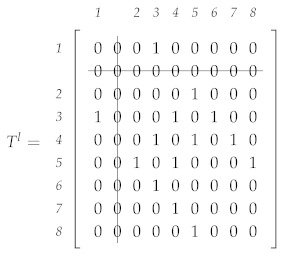


It can be noticed that the indexes of elements in a new matrix do not correspond to the agents’ identification numbers, except for agent 1. For instance, the adjacency matrix elements index for the agent with identification number 3 is equal to 2. For this reason, an identification vector *M* has been introduced. For the given example, the identification vector after failure of agent 2 is equal to

$$M^{l}=\begin{array}{c} 1\\ 2\\ 3\\ 4\\ 5\\ 6\\ 7\\ 8 \end{array}\left[\begin{array}{c} 1\\ 3\\ 4\\ 5\\ 6\\ 7\\ 8\\ 9 \end{array}\right]$$

In the demonstrated simple scenario, we have presented the limitations of connectivity maintenance when an agent fails. To overcome these challenges, we have proposed an algorithm for removing a critical agent from a cooperation multi-agent system. Algorithm 1 summarizes the strategy for determining a critical agent in the systems based on a local estimate of the algebraic connectivity and node degree. When a critical agent is detected, the algorithm removes a row and column from the agent’s local estimate of the adjacency matrix and updates the agent’s identification matrix. The algorithm is executed distributively on each agent *l* in the system and requires the following input variables: local estimate of the algebraic connectivity $\lambda_{2}^{l}\left(k-1\right)$, adjacency matrix $A^{l}\left(k-1\right)$, and identification vector $M^{l}\left(k-1\right)$. The algorithm calculates a new adjacency matrix $A^{l}\left(k\right)$ and identification vector $M^{l}\left(k\right)$.

The second possible scenario in a dynamically changing cooperating multi-agent system is adding a new agent in the system during task preformation. Consider a multi-agent system with an implemented decentralized connectivity maintenance strategy, and suppose that in those systems, a method for preserving the connectivity of the communication network is used. However, to the best of our knowledge connectivity maintenance algorithms are based on a fixed number of agents. To overcome the presented limitation in this section, we have proposed an algorithm for extending the local adjacency matrix and corresponding identification vector.
**Algorithm 1:** Removing a critical agent from the system.
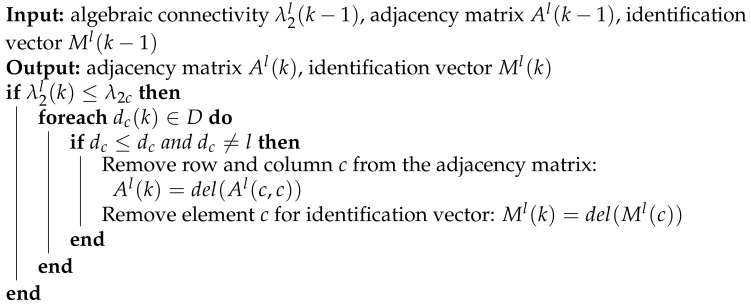


The procedure of adding an agent to an existing system starts with its ability to establish communication links with one or more agents in the existing system. If agent *m*, which is not a part of the system, is within a communication radius, it can initiate an adding procedure with one of its neighbours. The responsibility of the agent that receives the request is to check the ability of the new agent. We defined the ability of an agent as a sum of the links’ weights or the agent’s degree. In the other words, if the agent’s degree $d_{m}$ is not greater than the minimal required constant $d_{min}$, this means that its performance does not satisfy the minimal control quality of the operating system and is not an acceptable candidate for enlarging the system. On the contrary, when a new agent satisfies the minimal node degree requirement, it initiates an adding procedure and initializes its local estimate of the adjacency matrix elements and identification vector to the values of the neighbouring agent. After initialization, the new agent continues to compute is local estimate of the adjacency matrix by using the trust-based consensus algorithm described in Section 3.

When a new agent is an appropriate candidate for an existing network of cooperating agents, all agents in the system should add the new agent to their local estimates. Here, we defined two categories which have different sequences for adding a new agent. The first category includes all agents that are in the set of neighbours of a new agent *m*. Thus, if agent *l* is within a set of neighbours of agent *m*, or it holds $l\in N_{m}$, the following sequence is conducted:

1. Add agent *m* into the set of neighbours $N_{l}=\left\{N_{l},ID_{m}\right\}$.
2. Enlarge the identification vector $M^{l}$ with the address of the new agent *m*.
3. Enlarge the adjacency matrix $A^{l}$ with a row and column that correspond to the new agent *m*.

The second category includes all other agents that are more than one step away from the new agent. For each agent *l* that is not within the set of neighbours $l\notin N_{m}$, its local identification vector and adjacency matrix should be synchronized to a new size of the cooperating group. To achieve this goal, in the proposed algorithm, the following steps are conducted:

1. Agent *l* compares the size of the identification vectors with each agent in its set of neighbours $N_{l}$ .
2. If the size of the agent’s *l* identification vector is lower than the size of some agent from its neighbourhood, then the agent *l* should determine the address of new agent *m*.
3. If the degree of the new agent *m* is greater than the required threshold, then the adjacency matrix and identification vector of agent *l* are enlarged with values that correspond to the new agent.

The described procedure of adding one or more agents to the system in a decentralized way is summarized in Algorithm 2. Note that in the general case, if agent *l* does not have any neighbours, or it holds that $N_{l}=\emptyset$, this means that it is not a part of the multi-agent system. When agent *l* is not a part of a group, it should continuously try to establish communication links with agents that are eventually present in its communication range, and it initiates an adding procedure if it increases its node degree to a certain level.

**Algorithm 2:** Add a new agent to the system.

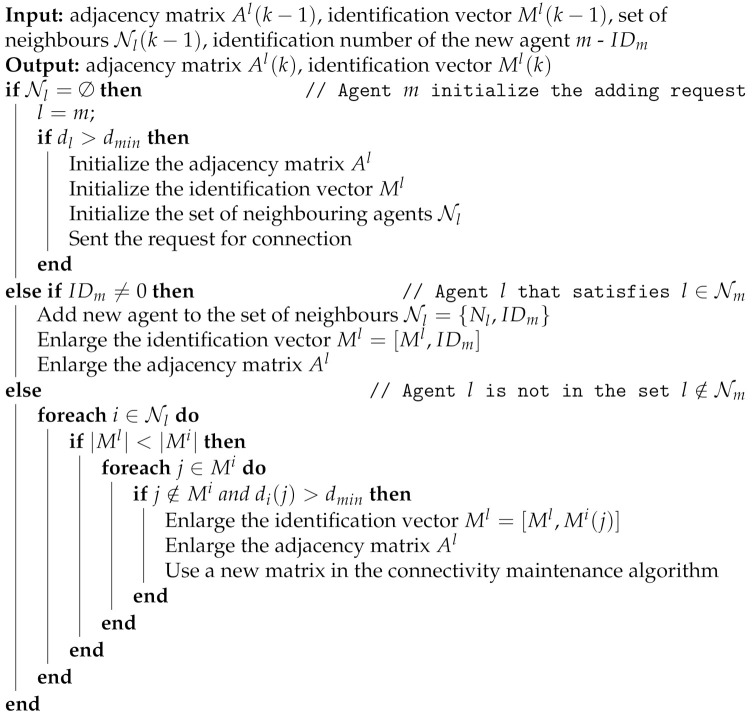



In the remainder of this section, we demonstrate the principle of the proposed algorithm on a system with eight agents. The agent’s *l* adjacency matrix $T^{l}$ and identification vector $M^{l}$ are equal to

$$\begin{array}{cc} \; & \;\;\;\;\;\;1\;2\;\;3\;\;\;\;4\;\;\;\;5\;\;6\;\;7\;\;8\;\\ \; & T^{l}=\begin{array}{c} 1\\ 2\\ 3\\ 4\\ 5\\ 6\\ 7\\ 8 \end{array}\left[\begin{array}{cccccccc} 0 & 0 & 1 & 0 & 0 & 0 & 0 & 0\\ 0 & 0 & 0 & 0 & 1 & 0 & 0 & 0\\ 1 & 0 & 0 & 1 & 0 & 1 & 0 & 0\\ 0 & 0 & 1 & 0 & 1 & 0 & 1 & 0\\ 0 & 1 & 0 & 1 & 0 & 0 & 0 & 1\\ 0 & 0 & 1 & 0 & 0 & 0 & 0 & 0\\ 0 & 0 & 0 & 1 & 0 & 0 & 0 & 0\\ 0 & 0 & 0 & 0 & 1 & 0 & 0 & 0 \end{array}\right] \end{array}$$

$$M^{l}=\begin{array}{c} 1\\ 2\\ 3\\ 4\\ 5\\ 6\\ 7\\ 8 \end{array}\left[\begin{array}{c} 1\\ 3\\ 4\\ 5\\ 6\\ 7\\ 8\\ 9 \end{array}\right]$$

In Figure 4, a network topology of the multi-agent system with nine agents is presented, where agent 2 is added to a group of eight agents. Dashed lines represent links established by agent 2 before sending an adding request. Therefore, agent 2’s set of neighbours is equal to $N_{2}=\left\{1,3,5\right\}$.

For the purpose of this example, suppose that agent 2 has ideal communication links whose weights are equal to 1. When its node degree is greater than the required threshold, it begins the adding procedure by sending a request to its neighbours and initializes its local estimates using values exchanged with its neighbours and local measurements. Accordingly, agent 2’s adjacency matrix and identification vector are equal to

$$\begin{array}{cc} \; & \;\;\;\;\;\;1\;2\;\;3\;\;\;\;4\;\;\;\;5\;\;6\;\;7\;\;8\;\;9\;\\ \; & T^{2}=\begin{array}{c} 1\\ 2\\ 3\\ 4\\ 5\\ 6\\ 7\\ 8\\ 9 \end{array}\left[\begin{array}{ccccccccc} 0 & 0 & 1 & 0 & 0 & 0 & 0 & 0 & 1\\ 0 & 0 & 0 & 0 & 1 & 0 & 0 & 0 & 1\\ 1 & 0 & 0 & 1 & 0 & 1 & 0 & 0 & 0\\ 0 & 0 & 1 & 0 & 1 & 0 & 1 & 0 & 1\\ 0 & 1 & 0 & 1 & 0 & 0 & 0 & 1 & 0\\ 0 & 0 & 1 & 0 & 0 & 0 & 0 & 0 & 0\\ 0 & 0 & 0 & 1 & 0 & 0 & 0 & 0 & 0\\ 0 & 0 & 0 & 0 & 1 & 0 & 0 & 0 & 0\\ 1 & 1 & 0 & 1 & 0 & 0 & 0 & 0 & 0 \end{array}\right] \end{array}$$

$$M^{2}=\begin{array}{c} 1\\ 2\\ 3\\ 4\\ 5\\ 6\\ 7\\ 8\\ 9 \end{array}\left[\begin{array}{c} 1\\ 3\\ 4\\ 5\\ 6\\ 7\\ 8\\ 9\\ 2 \end{array}\right]$$

Following the procedure of the algorithm, the local estimates of agents 1, 3, and 5 will be enlarged by the measurement of agent 2. Since the proposed algorithm is executed in a distributed manner, a new agent will be added to a local estimate of each agent on different time steps. The consequence of a distributed approach will be of a different order of identification vector and adjacency matrix elements when multiple agents are added in the same time period. However, this does not influence the final result of the algebraic connectivity estimate because of the described method of referencing the elements of the agent’s local adjacency matrix.

## 5. Results

**Figure 5:**
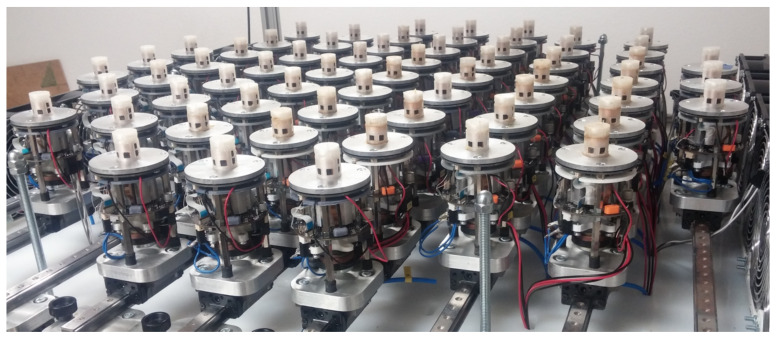
Multi-robot system for interacting with honeybees containing 61 independent devices called CASUs (combined actuator sensor units) [6].

The proposed algorithms for combining multiple communication channels and algebraic connectivity control in dynamic networked multi-agent systems have been experimentally verified, first in simulations, then in an experimental environment. The simulations, carried out in *MATLAB*, have been designed with the real multi-agent system described in [6] and presented in Figure 5 in mind. In the simulation environment, in which the agent’s operational principles are extracted from the *ASSISI Playground* platform introduced in [50], different scenarios have been evaluated using a relatively small number of agents (because of the presentation purposes). In Section 5.1, the proposed idea of combining multiple channels into a resulting communication network is evaluated. Section 5.2 provides the simulation results of the time responses of the graph’s algebraic connectivity when the number of agents in the collaborating group is changing. Finally, in Section 5.3, the algorithms proposed in this paper are experimentally verified using the developed multi-robot system comprised of 61 independent robots, called CASUs (combined actuator sensor units).

### 5.1. Multi-Channel Communication

In this experiment, we combine two different communication protocols, denoted as channel A and B, the performance of which has been embodied in the following quality constants:

- $K_{c}^{A}=1$;
- $K_{c}^{B}=0.9$.

**Figure 6:**
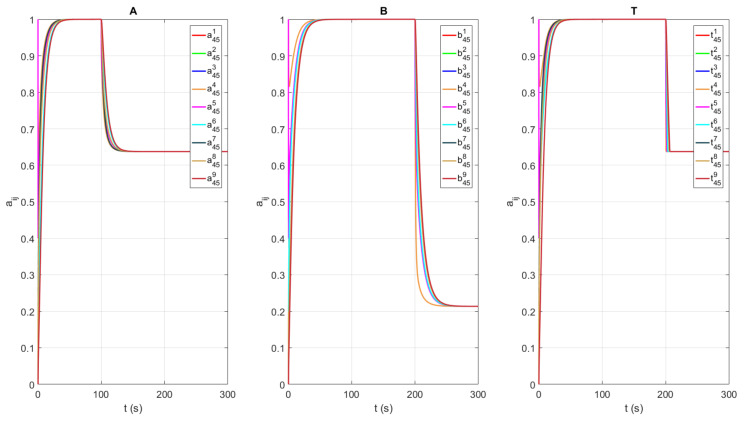
Adjacency matrix element (4,5) responses for channels A, B, and T.

We consider different network topologies for channels A and B as presented in Figure 1 and Figure 2, respectively. The experimental verification of multi-channel communication was carried out by changing the weight of the communication link between agents 4 and 5, or, in our case, the difference between the transmitted and received numbers of packages $\delta_{45}^{A}$ for channel A and $\delta_{45}^{B}$ for channel B. Table 1 summarizes the values of $\delta_{45}$ with respect to time *t*. The intervals have been chosen in a manner to capture three specific scenarios. The first scenario presents a case when both channels have the maximum performance, i.e., there is no difference between transmitted and received data. The second scenario describes the situation when the quality of channel A drops below the quality of channel B, i.e., $\delta_{45}^{A}<\delta_{45}^{B}$. Finally, the third scenario considers the case when channel A again has better quality than channel B, or it holds that $\delta_{45}^{A}<\delta_{45}^{B}$. Figure 6 shows the responses of agents’ local adjacency matrix elements (4,5) of channels A, B, and T.

**Figure 7:**
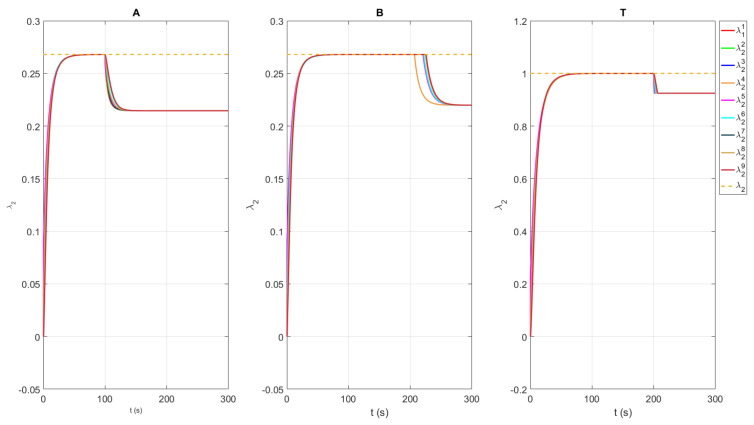
The responses for estimated algebraic connectivity for graph topologies defined with channels A, B, and T. Dashed line represents maximal value of the algebraic connectivity obtained in the case when all links in the considered graph’s topology exist.

Based on the estimated local adjacency matrix, each agent *l* calculates its local algebraic connectivity value, denoted as $\lambda_{2}^{l}$. The local estimate of the algebraic connectivity of each agent for graphs A, B, and T is depicted in Figure 7, where the dashed line represents the maximal value of the algebraic connectivity obtained in the case when all links in the considered graph topology exist. It can be observed that the maximal value of the algebraic connectivity for graph A is $\lambda_{2max}^{A}=0.26$, for graph B is $\lambda_{2max}^{B}=0.26$, and for the resulting graph T is $\lambda_{2max}^{T}=1$. Therefore, the value of the algebraic connectivity of the resulting graph T is almost 4 times greater than that from graphs A and B. The reason for this is that the resulting graph T combines links with better quality from both graphs. In other words, graph T is the union of graphs A and B. In the conducted experiment, the value of the algebraic connectivity estimate of graphs A and B depends on the link weight between agents 4 and 5, i.e., $\delta_{45}^{A}$ and $\delta_{45}^{B}$. The proposed algorithm is constantly optimizing the usage of available links in the resulting graph T in order to maintain the required value of algebraic connectivity. The robustness of the algorithm has been shown in the conducted experiment, where the algebraic connectivity value of the resulting graph T does not change until t>200 s.

### 5.2. Time Variable Multi-Agent System

**Figure 8:**
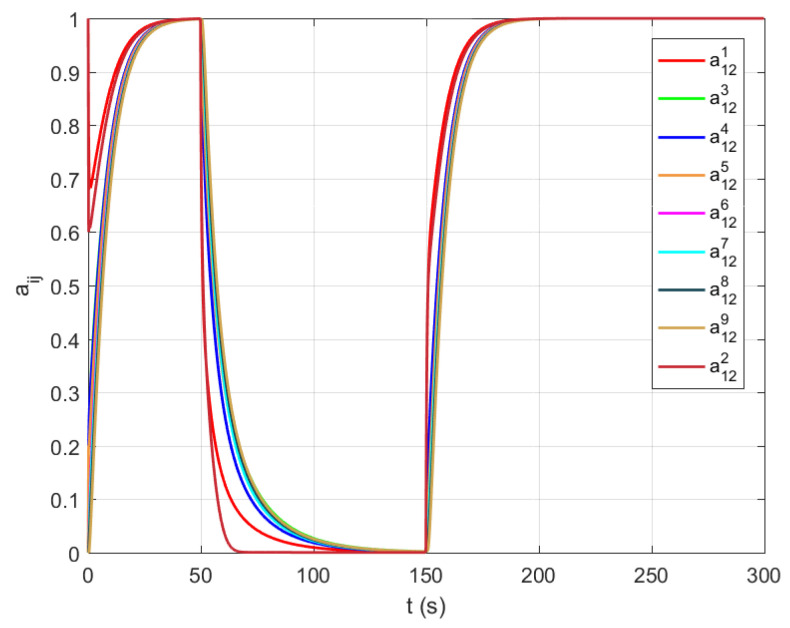
Time responses of the link $a_{12}$ weight on all agents in the system. At t=50 s agent 2 fails and recovers at t=150 s.

**Figure 9:**
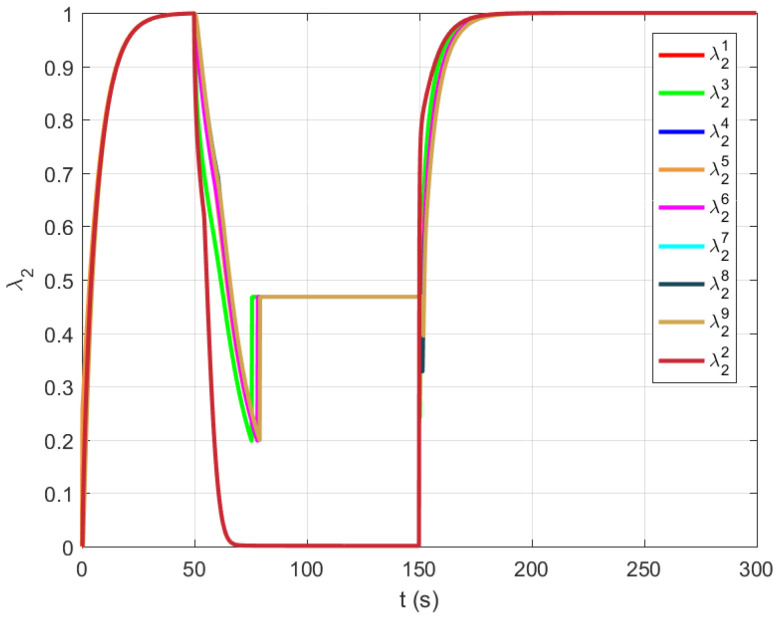
Time responses of the algebraic connectivity local estimate of all agents in the system.

In this section, we performed experimental verification of the proposed algorithms for maintaining the algebraic connectivity in a multi-agent system. The simulation is performed using a multi-agent system whose initial topology at t=0 s is presented in Figure 3 and Figure 4. We simulated a failure and recovery of agent 2. At time t=50 s, agent 2 fails and recovers at t=150 s, as shown in time responses of the link $a_{12}$ weight on all agents (Figure 8). The objective of the proposed algorithm is to detect and remove all agents without satisfactory quality of communication from a group decision-making process in the multi-agent system. Figure 9 presents the time responses of the algebraic connectivity local estimate of all agents in the system. The algorithm detects that agent 2’s algebraic connectivity and node degree are below the required minimal values and decides to disconnect agent 2 at approximately t=75 s.

Agent 2 recovers from the failure at time t=200 s by creating communication links with its fist neighbours. All agents in the systems detect a new agent and enlarge their estimated adjacency matrices. After a transient time, the local estimates of the algebraic connectivity converge to a common value that is equal to the value before failure.

### 5.3. Experimental Results

Experimental verification was performed in a real multi-robot system containing 61 CASUs. In particular, units inside this experimental setup do not have the possibility to change their positions during execution of the experiment. They are initially positioned inside a square area of 1 by 1 m, where each unit is able to communicate with its neighbouring units. The neighbouring set of each unit is unknown before the start of task execution and depends on the unit’s initial position and chosen communication protocol. The advantage of the chosen experimental setup is developed by two different types of communication protocols, i.e., Ethernet and infrared communication protocols.

**Figure 10:**
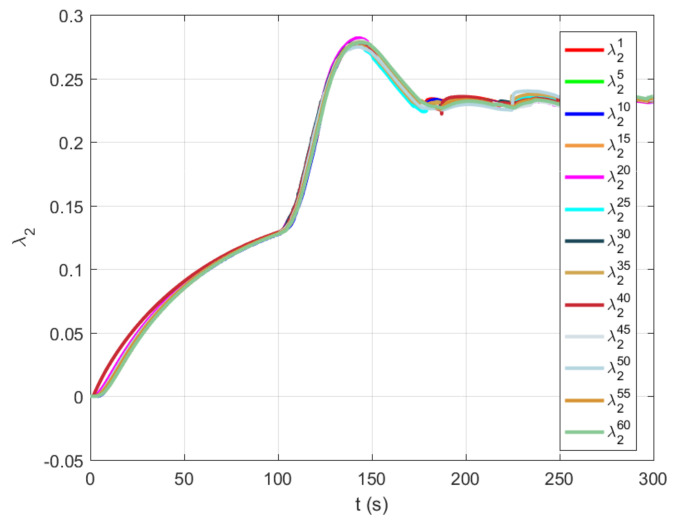
The responses for the algebraic connectivity for every fifth agent during experimental verification using 61 CASUs.

The algebraic connectivity for every fifth agent was presented in Figure 10. At the beginning of the experiment, agents first estimate the graph topology of the underlying network. The effect can be noticed until t=100 s, where a collective decision about the value of the algebraic connectivity of the communication graph converges to a common value. A new agent joined the group at time t=100 s, after which the algebraic connection of the group increased from $\lambda_{2}^{i}=0.13$ to $\lambda_{2}^{i}=0.24$. It can be noticed that the maximal value of the algebraic connectivity before reaching the stationary value of approximately $\lambda_{2}^{i}=0.24$ at the end of the experiment was $\lambda_{2}^{i}=0.28$. The main reason for the described overshoot is because a new unit does not have information about graph topology. Therefore, the connectivity maintenance algorithm wants to achieve the desired value of the algebraic connectivity in a short period.

Compared to the experiments conducted with nine agents, the convergence speed of the algorithm is much slower, which is a result of the different topology used in this experiment.

## 6. Discussion

As shown in [51,52], in multi-agent applications where the communication network is based on the agent’s onboard capabilities, connectivity control between agents is necessary to guarantee the system’s performance and achievement of the global goal. Several different approaches to preserve connectivity have been exploited, including approaches based on the algebraic connectivity of the underlying graph, methods that control the distance between neighbouring agents, and probabilistic models. Herein, we demonstrated that by introducing multiple communication channels, it is possible to control the value of the algebraic connectivity without the need for adding new links. In the obtained simulation results, two different scenarios were tested. The first scenario, presented in Section 5.1, demonstrates the implications of using multiple communication channels on the algebraic connectivity. With the proposed method, each agent in a distributed multi-agent system selects links whose qualities are the best among its set of neighbours. The second scenario, presented in Section 5.2, shows the impact of the agent’s failure or recovery to the connectivity measure. In simulations after a failed agent is no longer included in the estimation of the algebraic connectivity, its value is calculated for a graph that contains the remaining agents, as has been shown in Figure 3. Furthermore, the algebraic connectivity increases after node failure. This feature is the basic purpose of the proposed algorithm for controlling the number of nodes in a distributed ad hoc network. Since the algorithm is executed in a decentralized way, note that agents that are closer to the failed agent in the graph topology detect its inability to cooperate earlier.

In addition, preliminary experimental verification has been carried out in a real multi-robot system which contains 61 robots. The presented results show the scalability and effectiveness of the proposed distributed method for combining multiple communication channels and an algorithm that dynamically determines the collaboration group size.

To the best of our knowledge, a general approach that combines multiple communication protocols to create a reliable and resilient distributed network commonly used in applications such as sensor networks and multi-agent systems has not yet been presented in the literature. In the described distributed network, a communication graph becomes tolerant to variations in the link’s quality and the failures of agents. Consequently, agents that remain active are able to perform a given task. Since the computational complexity of the consensus algorithm is dependent on the number of agents in the network, future works will thoroughly investigate the influence of group size on the speed of convergence of the algebraic connectivity value in different real experimental setups.

## Figures and Tables

**Table 1:** The difference between transmitted and received number of packages for the links between agents 4 and 5 for channels A and B.

t [s]	[0,100〉	[100,200〉	[200,300]
Channel A ($\delta_{45}^{A}$)	0	0.3	0.3
Channel B ($\delta_{45}^{B}$)	0	0	0.5

## Data Availability

Not applicable.
